# Case Report: Evolution of a BRAF V600E-mutant papillary thyroid carcinoma from latissimus dorsi metastasis and squamous transformation to PD-L1 upregulation and effective immunotherapy

**DOI:** 10.3389/fimmu.2026.1762050

**Published:** 2026-04-29

**Authors:** Dan Tian, Shuqin Deng, Gaowa Jin, Lenggaowa Da, Wenjuan Wang, Yungaowa Wu, Jiali Gao, Ting Xu, Kexin Wei, Yalong Han, Jun Zhao, Quanfu Li

**Affiliations:** Department of Oncology, Ordos Central Hospital, Ordos, China

**Keywords:** BRAF V600E, latissimus dorsi metastasis, papillary thyroid carcinoma, PD-L1, squamous cell carcinoma

## Abstract

This case report describes an unusual clinical course of a patient with papillary thyroid carcinoma (PTC). The patient sequentially developed metastases to the cervical lymph nodes, lungs, pleura, and brain. Following tumor invasion into the latissimus dorsi muscle, a biopsy of this metastatic lesion revealed a BRAF V600E-mutant squamous cell carcinoma (SCC) phenotype indicating a high-grade dedifferentiation from the primary PTC. This metastatic focus also demonstrated upregulation of programmed cell death ligand 1 (PD-L1) expression. Subsequently, the patient exhibited a robust sensitivity to treatment with a programmed cell death protein 1 (PD-1) inhibitor and chemotherapy. This case highlights a highly aggressive evolutionary pattern of PTC and its associated potential for immunotherapy, providing valuable insights for the clinical management of thyroid cancer.

## 1 Introduction

Papillary thyroid carcinoma (PTC) is the most common well-differentiated endocrine malignancy, accounting for approximately 90% of cases (1). PTC is typically characterized as a low-grade invasive tumor, with distant metastases being relatively rare. The most common site of metastasis is the cervical lymph nodes, while a small number of cases may involve metastasis to the lungs, bones, or brain (2). Although most PTCs are well-differentiated with a low incidence of local invasion, recurrence, or metastasis, a minority of cases may exhibit tumor heterogeneity, presenting as more aggressive variants accompanied by distinct clinical, pathological, and molecular characteristics (3). Beyond oncogenic drivers, PTC progression and metastasis are critically shaped by the tumor microenvironment, particularly through immune evasion mediated by the programmed cell death protein 1 (PD-1)/programmed cell death ligand 1 (PD-L1) axis (4). This study reports a rare case of PTC in which the patient sequentially experienced metastases to the cervical lymph nodes, lungs, pleura, and brain, followed by a metastasis to the latissimus dorsi muscle. Notably, the metastatic lesion exhibited dedifferentiation with a squamous phenotype, while molecular testing on multiple samples showed the persistence of the BRAF V600E mutation. Concurrently, a striking upregulation of PD-L1 expression was observed via immunohistochemistry. Reporting this case aims to provide a reference for clinical practice.

## 2 Case description

**Figure 1:**
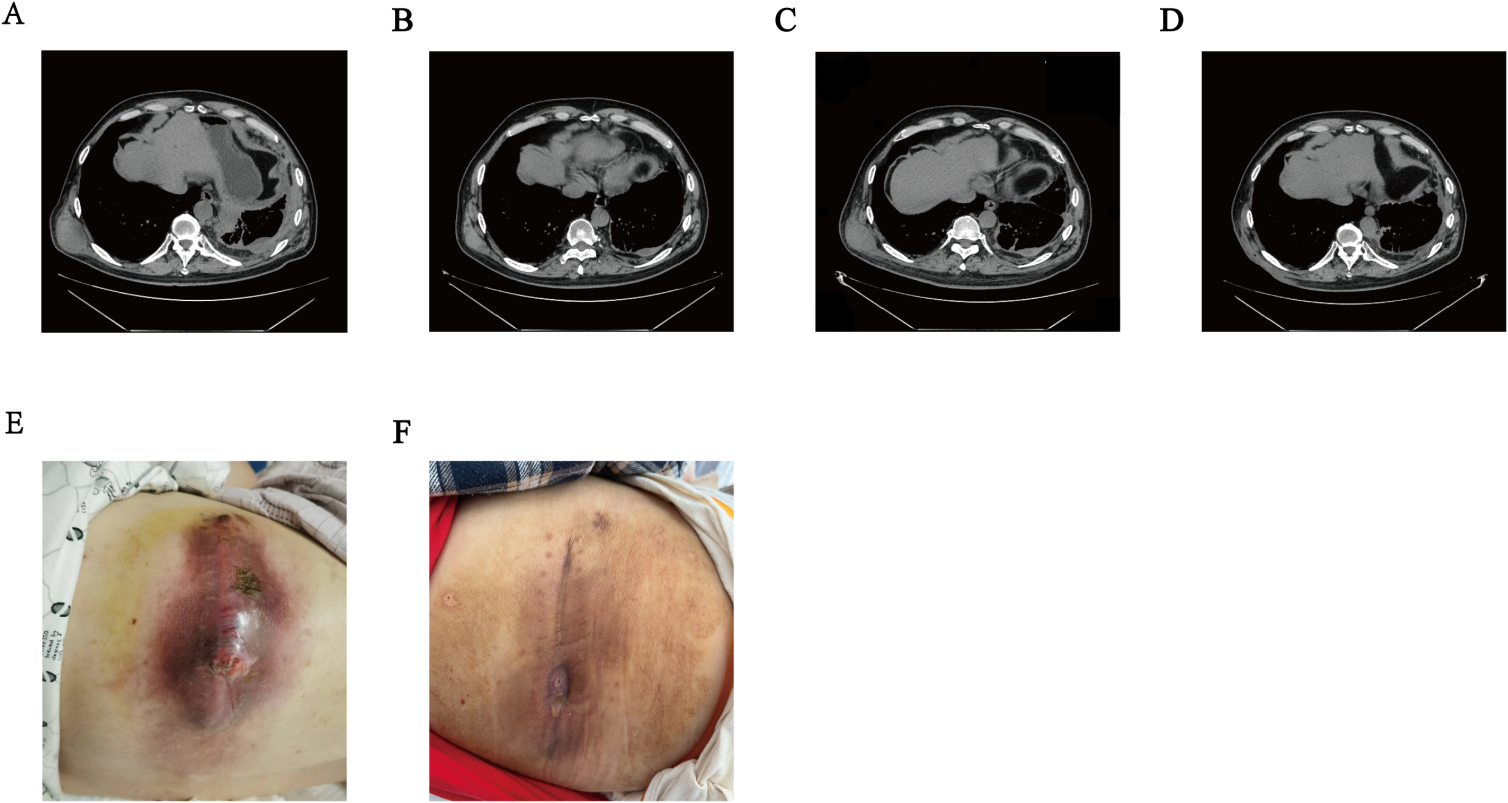
Changes in the patient’s latissimus dorsi metastasis throughout the treatment course: **(A)**. CT image from October 18, 2023, showing the initially identified latissimus dorsi metastasis. **(B)**. Shrinkage of the latissimus dorsi metastasis following treatment with dabrafenib combined with trametinib. **(C, E)**. CT image from October 18, 2024, showing further enlargement of the latissimus dorsi metastasis, along with a photograph of the skin invaded by the lesion. **(D, F)**. CT image and clinical photograph showing significant shrinkage of the latissimus dorsi metastasis after treatment with immunotherapy combined with chemotherapy.

**Figure 2:**
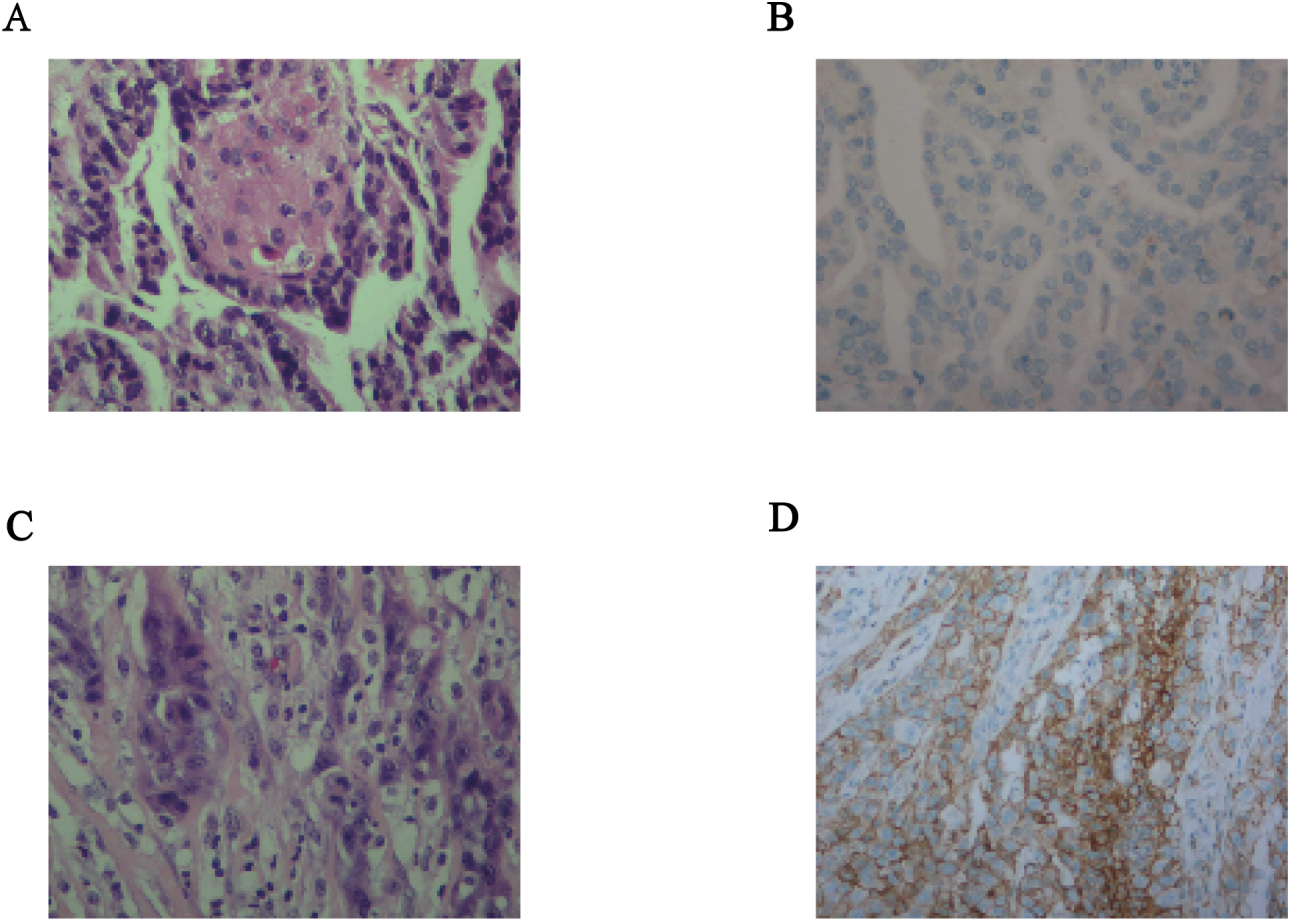
Pathological type of the patient’s latissimus dorsi metastasis and changes in PD-L1 expression by immunohistochemistry: **(A, B)**. Area of papillary thyroid carcinoma with squamous metaplasia (microscope was 400×), showing low PD-L1 expression at that time (CPS = 1) (microscope was 200×). **(C, D)**. Pathological examination of the latissimus dorsi lesion on October 18, 2024, revealing a squamous cell carcinoma (microscope was 400×) component with markedly high PD-L1 expression (CPS = 105) (microscope was 200×).

In July 2011, the patient inadvertently discovered a left neck mass. Ultrasound examination revealed a nodule in the thyroid isthmus, measuring approximately 1.5 x 0.8 cm, with enlarged lymph nodes in the right upper neck, bilateral mid-neck, bilateral tracheoesophageal grooves, and the anterior superior mediastinum. The findings were suggestive of a malignant thyroid tumor with cervical lymph node metastasis. In August 2011, the patient underwent a total thyroidectomy with bilateral lymph node dissection at the Cancer Hospital of the Chinese Academy of Medical Sciences. Intraoperative frozen section pathology indicated metastatic PTC in the lymph nodes, with no description of squamous metaplasia.Postoperatively, the patient received two courses of I-131 therapy. Follow-up examinations showed stable disease. The patient remained disease-free for 108 months until 2020, when multiple metastases were detected involving the cervical lymph nodes, lungs, pleura, and brain. A lung metastasis was confirmed via biopsy as metastatic papillary thyroid carcinoma.Subsequently, the patient underwent stereotactic radiotherapy (30 Gy/5 fractions/1 week) for the brain metastases. Concurrently, targeted therapy with sorafenib tosylate was initiated at a dose of 12 mg, administered orally once daily on days 1–14 of a 21-day cycle. The treatment response was evaluated as stable disease (SD). In October 2023, a follow-up chest CT scan revealed a mass in the patient’s right latissimus dorsi muscle (**Figure 1A**). The first progression-free survival (PFS1) duration had reached 37 months. The patient underwent the first needle biopsy of the latissimus dorsi mass. The pathological results indicated a metastatic papillary thyroid carcinoma focus, accompanied by squamous metaplasia (**Figure 2A**), suggesting potential pathological changes in the lesion. Immunohistochemical studies showed the following: CK(+),CK20 (–),TTF(+),CK5/6(squamous metaplastic areas+),P63(squamous metaplastic areas +),P40(squamous metaplastic areas +),PSA(-),NKx3.1(-),TG(-),CD56(+),D2-40(-),WT1(-),Calretinin(-),Ki67(+,5%),CK19(+),HBME1(+),NapsinA(-),PAX-8(+), p53 (wild-type),BRAF(+),PD-L1(22C3)(CPS=1),PD-L1(22C3Neg)(-) (**Figure 2B**). Next-generation sequencing (NGS) of the latissimus dorsi metastatic lesion identified a BRAF exon 15 mutation (p.V600E, c.1799T>A) with a variant allele frequency (VAF) of 13.2%. Consequently, starting in November 2023, the patient began treatment with dabrafenib 150 mg orally twice daily, combined with trametinib 2 mg orally once daily. The therapeutic response was evaluated as a partial response (PR) (**Figure 1B**). The second progression-free survival (PFS2) duration during follow-up was 12 months. By October 2024, the latissimus dorsi metastasis developed skin ulceration and exudation (**Figures 1C, E**). A third biopsy was performed. Pathological examination once again confirmed a metastatic papillary carcinoma of thyroid origin. Notably, in focal areas, tumor cells exhibited a solid growth pattern, and the immunohistochemical results supported the presence of a squamous cell carcinoma component (**Figure 2C**), indicating a significant pathological transformation of the tumor. The immunohistochemical results were as follows: PD-L1 (22C3) (CPS = 105), Ki-67 (70% in hot spots), P53 (focal high expression), TTF-1 (-), PAX-8 (+), Galectin-3 (+), HBME-1 (focal +), CK19 (+), BRAF V600E (+), CD56 (-), CK7 (+), CK20 (-), P40 (focal +), P63 (focal +), Thyroglobulin/TG (-), GATA-3 (-), ER (-), PR (-), P504S (partial weak +), RCC (-), Nkx3.1 (-), PSA (-).It is particularly noteworthy that the PD-L1 CPS value at this time was as high as 105 (**Figure 2D**). Repeat NGS of the latissimus dorsi metastatic lesion identified a BRAF exon 15 mutation (p.V600E, c.1799T>A) with a VAF of 33.92%, alongside a co-occurring PIK3CA exon 10 mutation (p.E545K, c.1633G>A) at a VAF of 13.6%. Considering the lack of approved indications for PIK3CA inhibitors in thyroid cancer and challenges regarding drug accessibility, a multidisciplinary team (MDT) consultation was convened. In light of the pathological features of the lesion, the treatment strategy was transitioned to a comprehensive modality combining immunotherapy and chemotherapy. The specific regimen is as follows: Sintilimab (200mg, iv, d1) in combined with chemotherapy (nab-paclitaxel 260 mg/m2, d1 and cisplatin 75 mg/m2, d1-2). After two cycles of treatment, the patient’s latissimus dorsi metastatic lesion showed significant shrinkage. According to the RECIST criteria, the therapeutic efficacy was evaluated as a partial response (PR) (**Figures 1D, F**; **Table 1**). Furthermore, the patient’s anxiety symptoms were markedly alleviated, which further improved treatment adherence and quality of life.

**Table 1:** Clinical timeline, pathological evolution, and therapeutic interventions (2011–2024).

Timepoint	Clinical status	Pathological &molecular findings	Therapeutic intervention	Clinical outcome
Aug 2011	Initial diagnosis: PTC with cervical lymph node metastasis	PTC	Total thyroidectomy + bilateral lymph node dissection; adjuvant I-131 therapy	DFS: 108 months
Sep 2020	First recurrence: multiple metastases (lungs, brain, pleura, cervical LNs)	Metastatic PTC	Stereotactic radiotherapy for brain; Sorafenib	PFS1: 37 months
Oct 2023	Disease progression: right latissimus dorsi mass	Metastatic PTC with squamous metaplasia; p53 (wild-type) PD-L1 CPS = 1 BRAF V600E (+) (VAF: 13.2%)	Dabrafenib + Trametinib	PFS2: 12 months
Oct 2024	Disease progression: ulcerated latissimus dorsi mass	SCC transformation (ATC); p53(+); PD-L1 CPS = 105 BRAF V600E (+) (VAF: 33.92%) PIK3CA (VAF: 13.6%)	Sintilimab + Nab-paclitaxel + Cisplatin	PR (Significant tumor shrinkage)

ATC, anaplastic thyroid carcinoma; CPS, combined positive score; DFS, disease-free survival; LNs, lymph nodes; PFS, progression-free survival; PR, partial response; PTC, papillary thyroid carcinoma; SCC, squamous cell carcinoma; VAF, variant allele frequency. Clinical response was evaluated according to the Response Evaluation Criteria in Solid Tumors (RECIST) version 1.1.

## 3 Discussion

PTC is generally an indolent disease with a 10-year survival rate of approximately 93% (3). While it is more frequently diagnosed in women aged 40–50 years, male patients often present with more aggressive features. This case report describes a male patient with a history of PTC who developed a rare metastasis to the latissimus dorsi muscle two years after the occurrence of multiple metastases.

Molecular genetic testing of this patient’s tumor specimen revealed a BRAF V600E mutation. BRAF gene mutations occur predominantly at the V600E codon and are found in approximately 60%-70% of PTC cases and 33%-40% of lethal anaplastic thyroid carcinoma (ATC) cases (5). BRAF encodes a serine/threonine kinase, which is a key component of the MAPK signaling pathway and is involved in cell proliferation, differentiation, and apoptosis. Abnormal activation of this pathway increases the risk of tumorigenesis (6).The BRAF V600E mutation has been associated with aggressive pathological features, including lymph node metastasis (LNM), advanced clinical stage, and poor prognosis (7).

In addition to the BRAF V600E mutation, a distinct phenotypic transformation was observed in the patient’s pathological profile. According to the latest WHO Classification, the squamous morphology that emerged in the later stages of this case should be categorized within the ATC spectrum rather than as a discrete pathological entity (8).At the molecular level, this malignant transformation was characterized by a shift from a p53 wild-type state with a low Ki-67 index (5%) in 2023 to a p53-mutant phenotype with a soaring Ki-67 index (70%) by 2024, accompanied by the complete loss of follicular differentiation markers (TTF-1/TG). The genomic evolutionary trajectory further substantiated this progression: the initial low-frequency BRAF V600E clone expanded into a dominant clone characterized by acquired TP53 and PIK3CA mutations. In alignment with the multi-step progression model proposed by Landa et al. (9), BRAF V600E established the genetic foundation, while the subsequent acquisition of TP53 and PIK3CA served as the core molecular catalysts driving dedifferentiation under therapeutic pressure. Despite the dismal prognosis typically associated with ATC, the patient sustained a PFS of 12 months following treatment with dabrafenib plus trametinib. This clinical outcome is highly consistent with landmark Phase II study data, which reported an ORR of 69% and a 12-month PFS rate of 79% (10). These findings underscore the pivotal value of precision targeted intervention in arresting the malignant dedifferentiation process of ATC.

However, it is noteworthy that despite treatment with the combination of BRAF and MEK inhibitors (BRAFi/MEKi), the patient developed resistance. The mechanisms of resistance to BRAF/MEK inhibitors can be categorized into RAF/MEK/ERK pathway-dependent and pathway-independent mechanisms. Among these, RAF/MEK/ERK pathway-dependent resistance mechanisms include: hyperactivation of receptor tyrosine kinases (RTKs), constitutive activation of RAS mutations, BRAF splice variants or copy number amplification, and MEK1/MEK2 mutations (6).Pathway-independent mechanisms primarily involve the loss of phosphatase and tensin homolog (PTEN), activation of the PI3K/AKT pathway, and persistent assembly of the eukaryotic translation initiation factor 4F (eIF4F) complex, among others (11).Based on the NGS results from October 2024, the patient’s acquired resistance may stem from the activation of the PI3K/AKT signaling pathway mediated by the PIK3CA (p.E545K) mutation. This suggests a potential bypass mechanism that overcomes BRAF inhibition, driving disease progression. Previous studies have suggested that this pathway may be involved in the transcriptional regulation of PD-L1 (12). Post-resistance, a pathological evolution from early squamous metaplasia to SCC was observed, temporally paralleled by a dramatic PD-L1 upregulation (CPS 1 to 105). To further elucidate the immunological landscape of this transformation, we assessed the density of tumor-infiltrating lymphocytes (TILs), which showed only a marginal increase from <1% to 1%–5%. This pronounced asymmetry—characterized by high PD-L1 expression alongside low TIL infiltration—suggests that the surge in PD-L1 was not a consequence of a robust adaptive immune response. Instead, it likely represents an intrinsic induction driven by the synergy of BRAF clone enrichment (13), PI3K/AKT activation (12), and the inherent molecular programming associated with squamous differentiation (14). Given that the squamous phenotype consistently exhibits high PD-L1 across various organs (15, 16), we propose that this upregulation serves as a phenotypic hallmark of adaptive tumor evolution and microenvironmental remodeling under therapeutic pressure (4), rather than a standalone indicator of immunotherapy efficacy. Taken together, these phenomena reflect a complex intersection of treatment-induced stress and genomic instability, which collectively orchestrated the progression toward a highly aggressive, dedifferentiated malignancy.

By integrating longitudinal clinical, pathological, and genomic evolution data, this case clearly elucidates the significant heterogeneity exhibited by a case of PTC under therapeutic pressure, as well as the molecular trajectory of its evolution from a well-differentiated state toward a highly aggressive, dedifferentiated phenotype. Several limitations of this case report must be acknowledged. First, as a single-patient study, our findings are primarily descriptive and warrant validation in larger cohorts. Second, while the detection of a PIK3CA mutation provided a plausible genomic explanation for BRAF-inhibitor resistance, the lack of more extensive multi-omic validation limits our understanding of the full molecular landscape driving this rapid phenotypic transition. Furthermore, since the patient received combined chemoimmunotherapy, the individual contributions of the PD-1 inhibitor and chemotherapy remain indistinguishable. The observed clinical benefit may stem from the synergistic effect of chemotherapy-induced immunogenic cell death and PD-1 blockade, or predominantly from a single modality. Consequently, this study is descriptive and precludes definitive causal inferences between therapeutic interventions and outcomes. Nevertheless, it highlights the critical importance of timely identifying phenotypic transformations and adjusting therapeutic strategies accordingly. While providing valuable insights for personalized medicine, these findings necessitate further validation in large-scale studies.

## Data Availability

The original contributions presented in the study are included in the article/supplementary material. Further inquiries can be directed to the corresponding authors.
